# PediTools Electronic Growth Chart Calculators: Applications in Clinical Care, Research, and Quality Improvement

**DOI:** 10.2196/16204

**Published:** 2020-01-30

**Authors:** Joseph H Chou, Sergei Roumiantsev, Rachana Singh

**Affiliations:** 1 Massachusetts General Hospital Boston, MA United States; 2 Harvard Medical School Boston, MA United States; 3 Baystate Children's Hospital Springfield, MA United States; 4 University of Massachusetts Medical School Springfield, MA United States

**Keywords:** growth charts, pediatrics, infant, newborn, infant, premature, failure to thrive, internet, software

## Abstract

**Background:**

Parameterization of pediatric growth charts allows precise quantitation of growth metrics that would be difficult or impossible with traditional paper charts. However, limited availability of growth chart calculators for use by clinicians and clinical researchers currently restricts broader application.

**Objective:**

The aim of this study was to assess the deployment of electronic calculators for growth charts using the lambda-mu-sigma (LMS) parameterization method, with examples of their utilization for patient care delivery, clinical research, and quality improvement projects.

**Methods:**

The publicly accessible PediTools website of clinical calculators was developed to allow LMS-based calculations on anthropometric measurements of individual patients. Similar calculations were applied in a retrospective study of a population of patients from 7 Massachusetts neonatal intensive care units (NICUs) to compare interhospital growth outcomes (change in weight Z-score from birth to discharge [∆Z weight]) and their association with gestational age at birth. At 1 hospital, a bundle of quality improvement interventions targeting improved growth was implemented, and the outcomes were assessed prospectively via monitoring of ∆Z weight pre- and postintervention.

**Results:**

The PediTools website was launched in January 2012, and as of June 2019, it received over 500,000 page views per month, with users from over 21 countries. A retrospective analysis of 7975 patients at 7 Massachusetts NICUs, born between 2006 and 2011, at 23 to 34 completed weeks gestation identified an overall ∆Z weight from birth to discharge of –0.81 (*P*<.001). However, the degree of ∆Z weight differed significantly by hospital, ranging from –0.56 to –1.05 (*P*<.001). Also identified was the association between inferior growth outcomes and lower gestational age at birth, as well as that the degree of association between ∆Z weight and gestation at birth also differed by hospital. At 1 hospital, implementing a bundle of interventions targeting growth resulted in a significant and sustained reduction in loss of weight Z-score from birth to discharge.

**Conclusions:**

LMS-based anthropometric measurement calculation tools on a public website have been widely utilized. Application in a retrospective clinical study on a large dataset demonstrated inferior growth at lower gestational age and interhospital variation in growth outcomes. Change in weight Z-score has potential utility as an outcome measure for monitoring clinical quality improvement. We also announce the release of open-source computer code written in R to allow other clinicians and clinical researchers to easily perform similar analyses.

## Introduction

### Background

Failure to thrive secondary to inadequate nutrition in the pediatric population may result in lifelong negative impact on physical and mental health outcomes [1,2]. This is especially critical for infants and children with known risk factors, such as preterm birth, acute and chronic illnesses, and social risk factors [3-7].

Anthropometric measurements commonly used in pediatric populations to assess nutritional status include weight, length, stature, head circumference, and midarm circumference. Using appropriate growth chart references, a single measurement alone indicates growth status for age at a single time point and may provide indications for closer monitoring. With multiple measurements, growth velocity over time can be evaluated and deviation from normal growth pattern may be suggestive of suboptimal nutrition or chronic illnesses, including metabolic disorders or congenital syndromes, although suboptimal monitoring itself may impact efficacy [8].

Before more widespread availability of electronic health records, paper growth charts were commonly used, but they had limitations, including infrequent updating, restricted accessibility for multiple care providers, and the inability to exactly determine percentiles numerically between the limited discrete percentile lines displayed on the printed charts.

The development of the lambda-mu-sigma (LMS) method for describing growth charts allows a quantitative description of growth charts based on tables of parameters [9]. In these tables, parameters for anthropometric measurements of interest relate a measurement at a given age to a precisely calculated Z-score (number of SDs from the mean) and percentile. Similarly, the expected anthropometric measurement at a particular Z-score and age can also be calculated. The availability of the LMS method and parameters for an increasing number of growth charts provides an opportunity to both improve clinical care of individual patients and allow large-scale analysis of datasets, which would be difficult or impossible if using paper growth charts.

Postnatal growth failure is common in preterm infants and is known to be associated with long-term neurodevelopmental impairment [10-18]. Extending the calculation of anthropometric measurement Z-scores from individual patients to a large population of patients might yield insight into how populations of preterm infants grow during their birth hospitalization. Similarly, we hypothesized that assessing the efficacy of quality improvement initiatives targeting improved growth might benefit from an unambiguous quantitative metric based on anthropometric Z-scores.

### Objectives

In this paper, we describe the deployment of the publicly accessible PediTools website, which implements a suite of calculators supporting LMS-based growth charts. We hypothesized that a simple metric to assess growth outcomes—the change in weight Z-score from birth to discharge (∆Z weight)—might yield insight into growth outcome variations. We retrospectively compared outcomes at 7 Massachusetts neonatal intensive care units (NICUs) and further utilized this metric to assess the efficacy of a nutrition-based quality improvement project at one of the NICUs. In addition, we also announce the release of open-source software, which will allow others to perform large-scale LMS-based calculations more easily.

## Methods

### Lambda-Mu-Sigma Method of Describing Growth References

The LMS method allows a parametric definition of growth references and generation of smoothed centile curves accounting for skewness of the distribution of an anthropometric measurement [9]. The parameters lambda (L, skewness normalization via power in the Box-Cox transformation), mu (M, mean), and sigma (S, coefficient of variation) describe the distribution of the measurement (eg, weight, length, or head circumference) at a given age, and the set of LMS parameters across multiple ages parameterizes the entire growth chart. This allows convenient calculation of exact Z scores (SDs from the mean) and generation of any centile curve.

#### Obtaining Lambda-Mu-Sigma Parameters

LMS parameters for growth charts were obtained either from the original publications, Web-based electronic supplements to the publications, and internet archives or by licensing agreement with the publication authors (references and sources listed in Table 1).

**Table 1:** Anthropometric growth calculators implemented on PediTools and sources of lambda-mu-sigma parameters.

Chart	Age range	Measures
Fenton 2003 preterm [19,20]	22-50 weeks gestation	Weight, head circumference, and length
Fenton 2013 preterm [21]	22-50 weeks gestation	Weight, head circumference, and length
CDC^a^ infant [22,23]	0-36 months	Weight, head circumference, and length
CDC pediatric [22,23]	24-240 months	Weight, height, and BMI
WHO^b^ infant [24]	0-24 months	Weight, head circumference, and length
Olsen preterm [25]	23-41 weeks gestation	Weight, head circumference, and length
WHO [26]	3-60 months	Arm circumference and triceps and subscapular skinfolds
CDC [27]	2-20 years	triceps and subscapular skinfolds
Olsen preterm BMI [28]	24-41 weeks gestation	BMI
Down syndrome infant [29,30]	0-36 months	Weight, length, and head circumference
Down syndrome pediatric [29,30]	2-20 years	Weight, height, head circumference, and BMI
CDC arm circumference [31]	2-222 months	Arm circumference
Mramba arm circumference [32]	60-228 months	Arm circumference

^a^CDC: Centers for Disease Control and Prevention.

^b^WHO: World Health Organization.

#### Interpolation of Lambda-Mu-Sigma Values

For each growth chart described via the LMS method, the L, M, and S curves are smoothed over ages, which permits interpolation of appropriate LMS values for intermediate values among the available discrete ages. In the PediTools calculators, simple linear interpolation was performed to obtain LMS values for intermediate ages. Different charts provide different degrees of age granularity. The Centers for Disease Control and Prevention (CDC) infant charts provide LMS parameters for ages in 1-month intervals, centered at the half-month point for the entire month [22], whereas the Fenton 2003 preterm charts provide parameters for completed weeks of gestation, centered midweek, for example, 30 weeks of completed gestation is centered around 30 3/7 weeks [19,20]. In contrast, the LMS values obtained for the Fenton 2013 preterm charts have values defined for each day of gestation; therefore, interpolation is not required [21].

#### Calculations via the Lambda-Mu-Sigma Method

**Figure 1:**
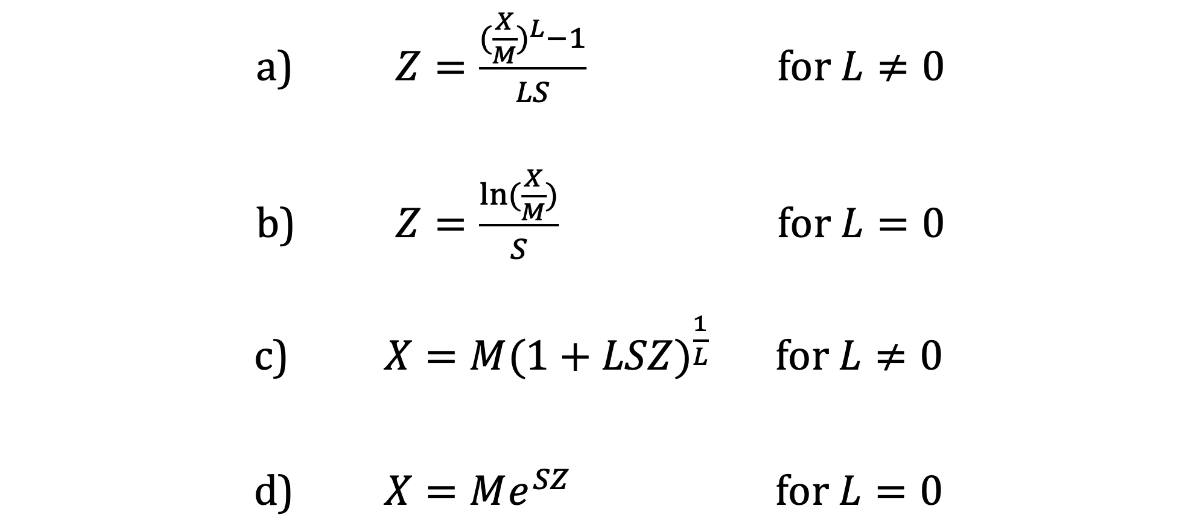
Equations for LMS-based growth metric calculations for Z score (a and b) and for an anthropometric measurement X (c and d).

Calculations of a Z score from LMS parameters and a given anthropometric measurement or an anthropometric measurement at a given Z score and LMS parameters at a particular age were performed as previously described (Figure 1) [9,22]. In the PediTools Web-based calculators, the percentile corresponding to a Z score was calculated by a numerical estimation of the cumulative density function of the standard normal distribution (equation 26.2.17 in the reference by Abramowitz et al) [33]. For the peditools R package, the same functionality is provided in the standard R function pnorm().

### PediTools Website

The PediTools Web calculators [34] were developed in PHP, a general-purpose scripting language well suited to Web development [35]. The website was generated using RapidWeaver version 7.5.7 (Realmac Software) [36]. Web hosting is currently provided by Bluehost Inc [37], under a shared hosting environment. Access statistics are tracked via Google Analytics. The PediTools Web server is configured to require the use of Secure Sockets Layer to encrypt traffic to and from the Web server. No data entered as inputs for the medical calculators are saved or analyzed.

For the PediTools Electronic Growth Chart, a Microsoft Excel spreadsheet template was designed to allow users to enter protected health information locally, but it would calculate the nonprotected health information values needed to generate a growth chart. Specifically, the date of birth, gender, gestational age at birth, and specific dates and measurements are entered, but only the gender and calculated postmenstrual ages and anthropometric measures are submitted to the PediTools electronic growth chart site, avoiding transmission of any specific dates.

For the PediTools electronic growth chart, LMS-based calculations were performed as above for all the measurements submitted. In addition, for sequential measurements, rate of weight change in grams per week, both observed and expected (to maintain the previous growth centile), were displayed.

As a visual aid to recognize excessive loss of weight Z-score between sequential measurements, after the first 10 days of life, the change in weight Z-score (∆Z) was color coded to display as red if the Z-score decreased by more than 0.06 SDs per week; yellow for decrease by more than 0.03 SD per week; and green otherwise. These thresholds were chosen somewhat arbitrarily, but over the course of a 14-week admission, each color would indicate an overall ∆Z weight of −0.84, −0.42, or less negative than −0.42 SDs, respectively.

### Multisite Comparison of Growth Outcomes

The Vermont Oxford Network (VON) is a nonprofit voluntary collaboration of neonatal health care professionals representing more than 1200 hospitals around the world [38]. Deidentified data were obtained from 7 level 3 NICUs in Massachusetts, which participate in the VON registry. Eligibility criteria included birth year from 2006 to 2011; gestational age between 23 0/7 and 34 6/7 weeks; no severe congenital malformations; and survival to discharge. Availability of birth weight, discharge weight, and length of stay were required to calculate the weight Z-score at birth and discharge. Infants were excluded if birth or discharge weight Z-scores were less than −4 or greater than 4, as values beyond these extremes often reflected data entry error. The calculated outcome metric was the ∆Z weight from birth to discharge. For NICU C, data for neonates born between 2012 and 2017 were also obtained for postintervention quality improvement outcomes analysis.

The VON registry provides a manual of operations with data definitions and eNICQ software, which allows for the collection, error checking, and submission of infant data. These manuals, data collection forms, and electronic data submission instructions are all available on the VON website. At each hospital, individual patient-level data for that hospital were exported from eNICQ as a CSV file, with 1 row per patient and 1 column per data field. (As of 2019, eNICQ data exports are now in XML or JSON format, but they contain identical information.) The data columns abstracted for each hospital in this study included the following: birth year (BYEAR), initial gestational age (GAWEEKS, GADAYS), birth weight (BWGT), length of stay (LOS1), and discharge weight (DWGT). Additional information obtained included source of admission (inborn or outborn, LOCATE), day of life of admission (DAYADMISS), discharge disposition (home, transfer to another facility, or death, FDISP), and congenital malformations (CMAL). Gender was not obtained, as at the time the study was originally conceived, the only preterm growth chart with LMS parameters available (Fenton 2003) was not gender specific [19]. Outcomes obtained but not reported here included the following: birth (BHEADCIR) and discharge (DHEADCIR) head circumference, early (EBSEPS) or late (LBPATH) bacterial infection, oxygen requirement at 36 weeks postmenstrual age (OX36), necrotizing enterocolitis (NEC, NECSURG), and retinopathy of prematurity (ISTAGE, ROPSURG).

Analysis was performed using R, free software for statistical computing [39], using the free version of the RStudio integrated development environment [40]. For data visualizations, smoothed conditional mean curves were generated by the R ggplot [41] package via generalized additive model and cubic splines [42]. When present, the bands surrounding the smoothed curves represent the 95% CI around the mean.

Comparisons among hospitals were performed by 2-tailed *t* test, analysis of variance, Wilcoxon rank-sum, and Kruskal-Wallis test, as appropriate. Multihospital ∆Z analysis was performed by fitting a linear model of ∆Z versus gestational age, with interaction terms for both slope and intercept for each hospital. When multiple pairwise comparisons were performed, multiple testing adjustment was performed by the Tukey honestly significant difference method. The study was approved by the Institutional Review Boards at each of the hospitals that contributed data.

### Single-Site Growth Outcomes Quality Improvement Project

Multiple bundled growth and nutrition quality improvement interventions were essentially simultaneously implemented at NICU C, starting in late 2011. These bundled changes included the following: (1) raised awareness of baseline growth failure by educational presentations to clinicians, showing how growth outcomes differed between NICU C and NICU F; (2) development of an electronic growth chart, as described in the PediTools Web tool; (3) systematic weekly growth metric collection in a form compatible with the electronic growth chart tool; (4) formal review of all NICU patients and their interval growth at weekly multidisciplinary rounds with pediatric dieticians; (5) earlier and broader initiation of parenteral nutrition with increased protein content and more rapid advancement; (6) revision of enteral feeding advancement protocols, including earlier initiation of gut priming (trophic feeds).

Assessment of the effect of the bundled interventions was performed similar to the analysis of growth outcomes described above: ∆Z weight from admission to discharge was calculated for each patient and the results were analyzed over different birth year epochs.

### Dissemination of Methods for Large-Scale Analysis

The R code used for the calculation of anthropometric measure Z scores from LMS parameters was bundled [43] into the R peditools package, and this will be hosted on GitHub [44] and shared under the Massachusetts Institute of Technology (MIT) License. The peditools package can be installed using the devtools package [45], with the command install_github(“jhchou/peditools”).

All growth charts described in this work are supported by the R peditools package, with the exception of the Fenton 2013 growth chart [21], for which the LMS parameters are available from the author by license only. If the Fenton 2013 parameters become publicly available in the future, they will be added to the peditools R package. In the meantime, the Olsen 2010 [25] or gender nonspecific Fenton 2003 [19] charts can be used to analyze preterm growth.

## Results

### PediTools Website

**Figure 2:**
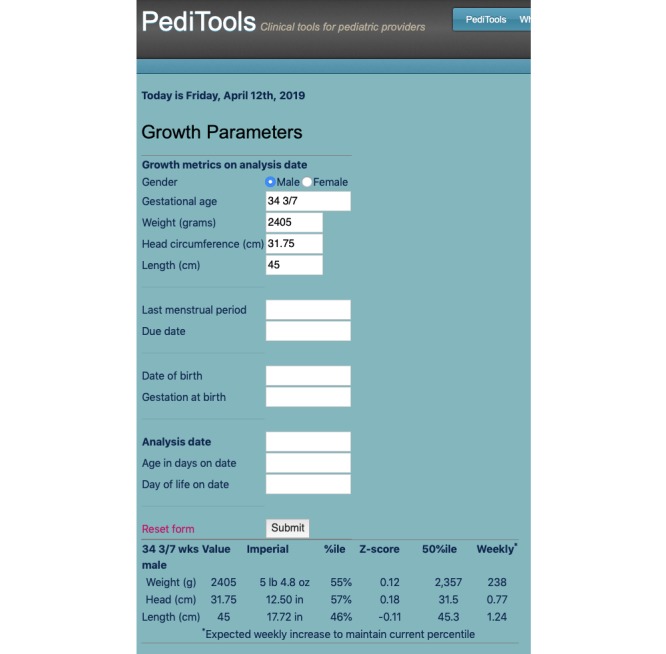
Screenshot of a representative PediTools web-based growth calculator (Fenton 2013 for preterm infants). The upper section demonstrates flexible support for multiple methods of input data entry. Data entry can include age as either gestational age or specific dates; measurements can be entered in metric or imperial units (grams or pounds and ounces; centimeters or inches); and even if no measurement is entered, the expected median (50th percentile) values will be displayed. The lower section displays the results of the LMS-based calculations, including the anthropometric measures in both metric and imperial units, percentile, Z-score, expected median measurement for age, and weekly growth required to maintain the current percentile.

The first PediTools Web calculator was developed in 2011 as an in-house tool to improve documentation of anthropometric measurements of premature newborns cared for at hospital C by allowing calculation of Z-scores and percentiles, using the Fenton 2003 preterm growth chart [19], for which LMS parameters were published in 2007 [20]. The webpage was moved to public hosting in January of 2012. A screenshot of a representative Web-based PediTools growth calculator is shown in Figure 2.

Although accessible to the general public, the target audience and purpose of the PediTools website are pediatric clinical providers’ bedside use. PediTools is agnostic to which growth charts are made available and does not provide recommendations as to which charts are appropriate for which populations. The users of the website are expected to exercise their own professional clinical judgment to determine suitability for their purposes.

Additional growth chart calculators have subsequently been added to PediTools, including support for the Fenton 2013 preterm chart [21], CDC infant and pediatric [22,23], World Health Organization (WHO) infant [24], Olsen 2010 preterm [25], WHO arm circumference and triceps and subscapular skinfold [26], CDC triceps and subscapular skinfold [27], Olsen 2015 BMI for preterm [28], Zemel 2015 Down syndrome [29,30], Abdel-Rahman 2017 midupper arm circumference [31], and Mramba 2017 midupper arm circumference [32].

As PediTools Web calculators were intended to be used by clinicians at the point of care, features in addition to reporting percentiles and Z-scores were integrated to promote ease of use and clinical relevance. For example, with the preterm calculators, a gestational age calculator was integrated to allow entry of either the postmenstrual gestational age of interest or any combination of last menstrual period, due date, delivery date, or chronologic age. For assessment of obesity, the CDC pediatric growth calculator includes both the Z score for BMI and updated categorization of extreme obesity, defined as BMI≥120% of the 95th percentile or ≥35 kg/m^2^ [46]. Both international and imperial units are supported. For infant calculators, calculations at both chronologic age and age corrected for prematurity can be reported, which is beneficial when assessing for timely attainment of developmental milestones. To help set goals for future growth, the calculators report the expected amount that anthropometric measures should increase over time to maintain the current Z-score (ie, equivalent to growing along the current percentile curve).

#### PediTools Website Utilization

**Figure 3:**
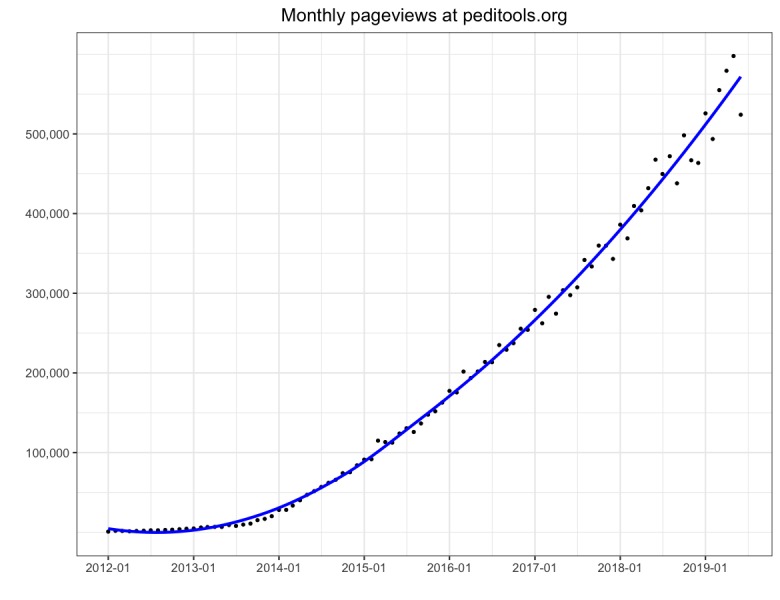
PediTools (https://peditools.org/) website overall monthly pageviews over time from public launch in January 2012 through June 2019.

The PediTools Web calculators have been well received by the clinical community. Since its public launch in January 2012, website access has increased to more than 500,000 page views per month. Figure 3 documents the increasing monthly page views over time; Table 2 shows page views by calculator for the year ending June 2019. Users were primarily from the United States (433,438/520,450; 83.28% users), but there were at least 1000 users from each of another 21 countries, with over 3000 users from Canada (17,169/520,450; 3.30%), India (5619/520,450; 1.08%), Australia (5096/520,450; 0.98%), Mexico (4066/520,450; 0.78%), and Brazil (3546/520,450; 0.68%). Access was primarily from desktop devices (307,326/518,796; 59.23%), followed by mobile devices (201,970/518,796; 38.93%) and tablets (9500/518,796; 1.83%).

PediTools also includes several aids not related to anthropometric measurements, including a bilirubin tool, which assists in the management of neonatal hyperbilirubinemia per the American Academy of Pediatrics 2004 guidelines [47,48] and a stand-alone version of the gestational age calculator, which is also incorporated in the preterm growth calculators. They will not be further discussed here, but they are mentioned as they receive the 4th and 6th largest number of page views, respectively.

**Table 2:** PediTools page views by calculator for year ending June 2019.

Web page views	Value (N=5,192,170), n (%)
Fenton 2013 preterm	1,438,367 (27.54)
CDC^a^ pediatric	1,338,920 (25.64)
WHO^b^ infant	954,634 (18.28)
Bilirubin tool	411,897 (7.89)
CDC infant	360,440 (6.94)
Gestational age tool	257,201 (4.92)
Olsen 2010 preterm	126,486 (2.42)
CDC mid-upper arm circ	79,654 (1.53)
Electronic growth chart	79,051 (1.51)
Down syndrome, infant	46,163 (0.88)
Olsen BMI preterm	35,396 (0.68)
Down syndrome, pediatric	28,214 (0.54)
WHO arm and skinfold	21,185 (0.41)
Fenton 2003 preterm	14,562 (0.28)

^a^CDC: Centers for Disease Control and Prevention.

^b^WHO: World Health Organization.

#### Electronic Growth Chart

**Figure 4:**
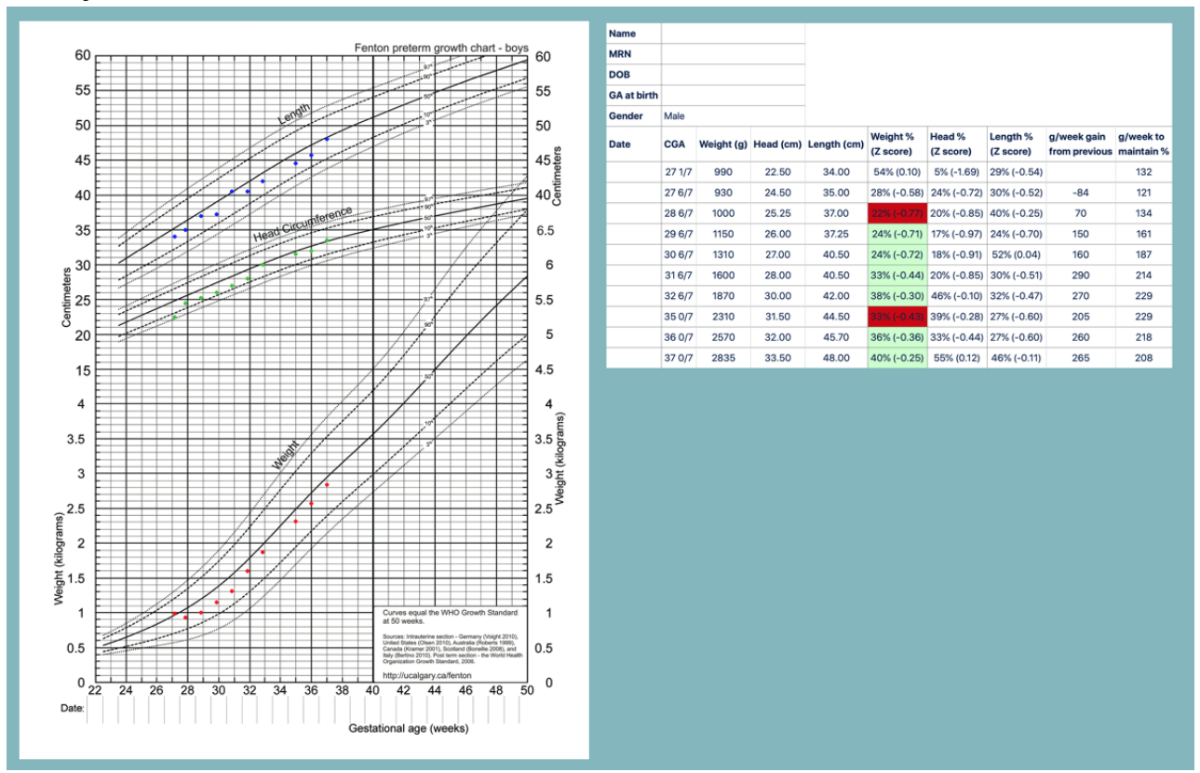
Electronic Fenton 2013 preterm growth chart. De-identified demographic and anthropometric data is copied into a webpage form from a specifically designed Microsoft Excel™ spreadsheet. The upper panel shows each anthropometric measurement plotted automatically onto the traditional paper-based chart. The lower panel displays calculated percentiles, Z-scores, and weekly weight change, both the actual observed change as well as the expected weekly change needed to maintain the previous percentile. Clinical decision support is provided by color-coding based on the weekly weight Z-score change.

The growth chart Web calculators on the PediTools website are limited in that only a single measurement can be analyzed at a time, whereas growth reflects how measurements change over time. For the Fenton 2013 preterm growth chart, an additional tool was developed to allow monitoring growth over time. As a public access website, care needs to be taken to not encourage sending protected health information over the internet. A Microsoft Excel spreadsheet was developed, in which specific dates and measures could be entered, but only deidentified data would be copied and pasted for secure submission via a webpage form. The output graphic was based on the original published chart [21] but with all the points plotted and supplemented with a table of percentiles, Z-scores, expected versus observed growth, and clinical decision support provided by color coding significant changes in Z-score between measurements (Figure 4). This tool was also used as part of a quality improvement project for longitudinal growth outcome monitoring (see below).

### Multisite Comparison of Growth Outcomes

#### Variation in Overall Growth Outcomes at Different Hospitals

Our first aim was to demonstrate the feasibility of using ∆Z to assess growth outcomes of premature newborns and to compare outcomes among hospitals. All infants born between 23 0/7 and 34 6/7 weeks gestational age from 2006 to 2011 at 7 level 3 NICUs in Massachusetts, with VON registry data available and who survived to discharge, were analyzed for growth outcomes analysis (Table 3). Weight Z-scores at birth and discharge and the change in Z-score from birth to discharge were calculated for each individual patient.

As shown in Table 3, the mean ∆Z from birth to discharge differed significantly by site (*P*<.001), with the overall mean ∆Z across all sites −0.81 and ranging across the 7 sites from −0.56 and −1.05.

**Table 3:** Study population of 7975 premature newborns born between 23 and 34 weeks of completed weeks gestation in 7 Massachusetts newborn intensive care units (A-G).

Metric	All NICUs^a^ combined (n=7975)	A (n=461)	B (n=1586)	C^b^ (n=1068)	D (n=418)	E (n=598)	F (n=1081)	G (n=2763)	*P* value
Gestational age (weeks), median (IQR)	32 (29.29-33.86)	29 (27.14-30.57)	33 (30.43-34.14)	32.86 (30.71-34.04)	31.86 (29-33.57)	29.14 (26.89-30.71)	31 (28.57-33.29)	32.57 (30.29-34)	<.001
Birth weight (gram), median (IQR)	1580 (1180-2050)	1130 (885-1316)	1810 (1370-2160)	1818.50 (1380-2195)	1462.50 (1100-1943)	1160 (871.25-1365)	1400 (1065-1860)	1750 (1340-2100)	<.001
Birth weight Z-score, mean (SD)	−0.19 (0.84)	−0.40 (0.85)	−0.14 (0.83)	−0.07 (0.81)	−0.32 (0.79)	−0.36 (0.91)	−0.24 (0.85)	−0.14 (0.82)	<.001
Discharge postmenstrual age (weeks), mean (SD)	36.4 (2.86)	35.71 (4.37)	36.64 (2.22)	36.17 (2.42)	34.88 (3.03)	36.95 (2.70)	37.05 (3.60)	36.31 (2.58)	<.001
Discharge weight Z-score, mean (SD)	−1.00 (0.80)	−1.15 (0.81)	−1.19 (0.77)	−0.88 (0.78)	−0.90 (0.76)	−1.32 (0.77)	−0.80 (0.80)	−0.93 (0.79)	<.001
Weight delta Z^c^, mean (SD)	−0.81 (0.52)	−0.75 (0.49)	−1.05 (0.49)	−0.80 (0.40)	−0.58 (0.46)	−0.96 (0.59)	−0.56 (0.52)	−0.79 (0.49)	<.001

^a^NICU: neonatal intensive care unit.

^b^An additional 1120 neonates born between 2012 and 2017 from NICU C were included for postintervention outcomes analysis, not tabulated here.

^c^Weight delta Z is the change in Z-score for weight from birth to discharge [19,20].

#### Correlation of Growth Failure With Gestational Age at Birth

**Figure 5:**
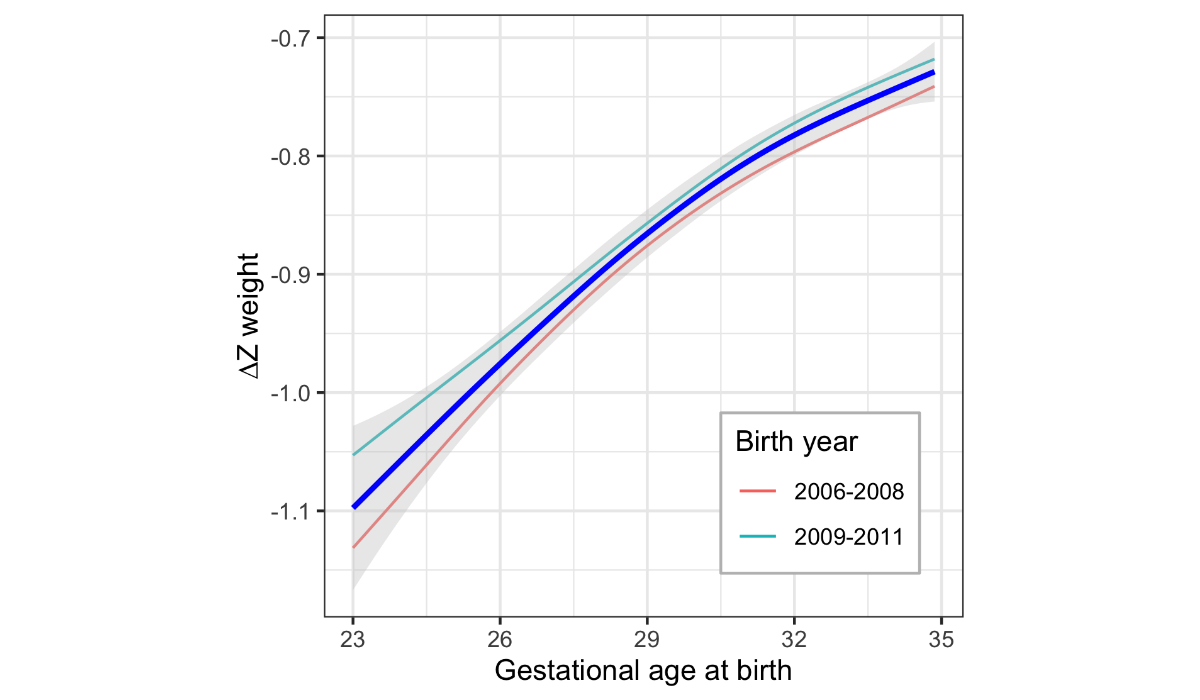
Change in weight Z-score from birth to discharge versus gestational age at birth, demonstrating inferior growth with increasing prematurity for all seven NICUs combined. The dark blue line is for all years 2006 - 2011 combined with the gray band representing the 95% confidence interval; the thin lines show the grouped birth years 2006 - 2008 versus 2009 - 2011.

Growth outcomes in the preterm population are potentially dependent on multiple factors, including gestational age at birth, nutrition practices, and timing of discharge. One of our hypotheses was that the ∆Z weight from birth to discharge might be associated with gestational age at birth. Combining data from all 7 hospitals across the entire time period from 2006 to 2011 and plotting the ∆Z weight versus gestational age at birth showed inferior growth (ie, more negative change in Z-score) with increasing prematurity (Figure 5). Grouping the data by birth year 2006 to 2008 and 2009 to 2011 showed that the relationship between more negative ∆Z weight and lower gestational ages appears unchanged over the 2 time epochs, although the shift in the lines upward suggests less loss in weight ∆Z score in the later epoch.

By visual inspection, the relationship between ∆Z weight and gestation at birth appears roughly linear. Fitting a linear regression allowed estimation of the relationship between growth failure and gestation at birth. At 29 0/7 weeks, the expected mean ∆Z weight from birth to discharge was −0.88 (*P*<.001, 95% CI −0.865 to −0.893), with each additional week of decrease in gestational age contributing an additional −0.029 (*P*<.001, 95% CI −0.025 to −0.033).

#### Interhospital Variation Between Growth Outcomes and Gestational Age at Birth

**Figure 6:**
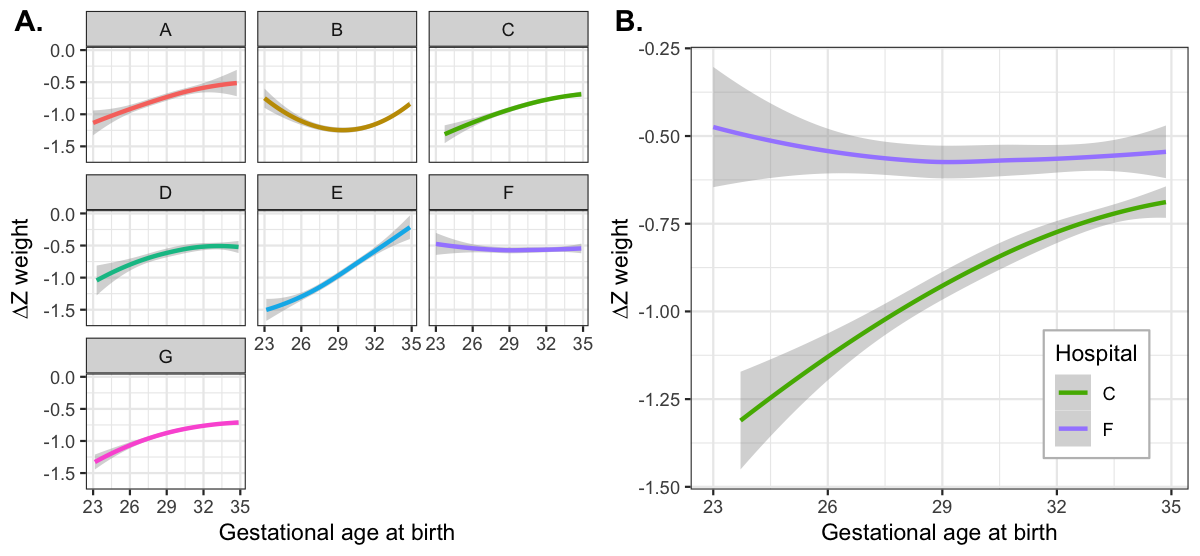
Inter-hospital variation in change in weight Z-score from birth to discharge, as related to gestational age at birth, (A) separately for each of seven different hospital NICUs in Massachusetts, and (B) for hospitals C and F overlaid on the same plot to better demonstrate inter-hospital differences.

It was possible that this inverse relationship between growth failure and gestational age at birth was intrinsic to prematurity and was therefore universal among the hospitals. To test this hypothesis, we next analyzed whether different hospitals might have different growth outcome characteristics. We found that the relationship between ∆Z weight and gestation at birth differed by hospital (Figure 6), with significant interhospital variation in both the degree of growth failure and the interaction with gestational age at birth. Some hospitals show much inferior growth at lower gestational ages at birth (eg, hospital E), whereas other hospitals show better growth overall and absence of inferior growth at lower gestational age (eg, hospital F). Patterns of growth at individual hospitals remained stable across different birth year epochs (data not shown), suggesting reliability for use as a quality improvement metric.

The differences can be seen clearly when choosing hospital F as the baseline hospital and comparing pairwise with every other hospital (eg, Figure 6). Fitting a linear model of ∆Z versus gestational age, with interaction terms for both slope and intercept for each hospital, demonstrated statistically significant differences between hospitals A-E and G compared with hospital F as baseline. Hospital F did not show a relationship between gestational age at birth and ∆Z weight (slope=−0.001, *P*=.85, 95% CI −0.010 to 0.009), and at 29 0/7 weeks, the mean ∆Z was −0.553 (*P*<.001, 95% CI −0.586 to −0.520). In comparison with hospital F, each of the other hospitals had both a more negative ∆Z at 29 0/7 weeks (all *P*<.003) and a greater relationship between increasing prematurity and more negative ∆Z (all *P*<.001).

In summary, we found that although all 7 hospitals studied had negative weight ∆Z from birth to discharge, hospitals differed in degree of negative ∆Z (ie, intercept at a gestational age of 29 0/7 weeks), as well as the degree to which inferior growth was related to lower gestational ages (ie, slope).

### Single-Site Growth Outcomes Quality Improvement Project

As poor growth trajectory might exacerbate long-term neurodevelopmental impairment, particularly for those patients born the most preterm, NICU C embarked on a multifocal quality improvement project to reduce the loss in weight Z-score from birth to discharge. Bundled interventions introduced in 2011 targeting factors potentially contributing to poor growth included the following:

- Utilized baseline data to raise awareness of poor growth, for example, in comparison with NICU F
- Implemented system of weekly growth metric collection
- Formal weekly multidisciplinary (including pediatric dietician) review of electronic growth chart
- Earlier and broader initiation of parenteral nutrition
- Increased protein content in premade parenteral nutrition
- Accelerated advancement of parenteral nutrition
- Earlier initiation of enteral nutrition
- Revised enteral feeding advancement protocol
- Pasteurized donor human milk made available

**Figure 7:**
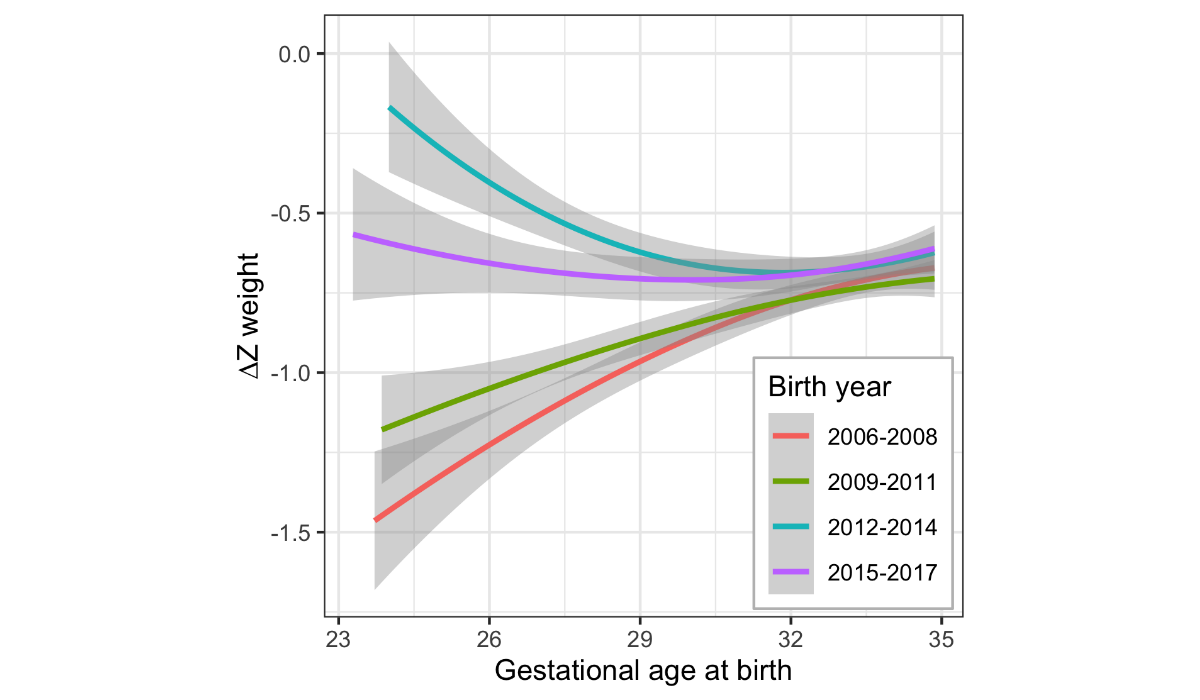
Improvement of growth outcomes (∆Z weight) at hospital C, by birth gestation and birth year epoch. Epoch 2006 – 2008 is pre-intervention; 2009 - 2011 covers the beginning of implementation of interventions; 2012 - 2014 is the immediate post-intervention epoch; 2015 - 2017 demonstrates sustained improvement, but less extreme at lower gestational ages, after targeting a goal ∆Z weight of -0.6. The largest improvements are seen at the lowest gestational ages at birth.

These interventions were associated with a significant reduction in loss of weight Z-score, particularly at the lowest gestational ages, which was sustained and progressive (Figure 7). Compared with the preintervention epoch 2006 to 2008, there was a marked reduction in loss of birth weight Z-score in the postintervention epoch from 2012 to 2014.

Using the 2006 to 2008 epoch as a reference and fitting a linear model of ∆Z weight versus gestational age at birth, with interaction terms for both slope and intercept (centered at 29 weeks) for the remaining 3 epochs of 2009 to 2011, 2012 to 2014, and 2015 to 2017, showed significant differences in both the ∆Z weight at 29 weeks (differences in intercept, *P*=.03, <.001, and <.001) and association with gestational age at birth (differences in slope, *P*=.015, <.001, and <.001, respectively).

In fact, because of concern that the degree of reduction of weight Z-score loss at the lowest gestational ages might be excessive in the 2012 to 2014 epoch, potentially contributing to future development of the metabolic syndrome [12], a less aggressive approach was adopted. The subsequent growth target was a ∆Z from birth to discharge of roughly −0.6 for all gestations, resulting in the more flattened curve of epoch 2015 to 2017 (Figure 7).

### Dissemination of Methods for Large-Scale Analysis

The PediTools website has met a need for clinicians wishing to analyze data for an individual patient at a time. In contrast, the multisite comparison of growth outcomes of thousands of patients at 7 NICUs yielded additional insight into growth patterns of preterm newborns and prompted a successful quality improvement project at 1 NICU. Here, we describe the release of open-source computer code to permit others to conveniently do similar analysis, which should be useful for both clinical research and quality improvement monitoring.

The peditools R package will be made available on GitHub and provide functions to work with LMS-based anthropometric charts, including all LMS parameters possible [44]. All growth charts available on the PediTools website (listed in Table 1), with the exception of the Fenton 2013 preterm growth chart, are included. At this time, the Fenton 2013 chart LMS parameters are available by license only and are restricted from being shared. As additional charts are added to the PediTools website, the peditools R package will also be updated to include the new charts. The peditools R package will be released under the relatively permissive MIT License, which allows for commercial use, modification, redistribution, and sublicensing.

The primary package tool is the peditools::x_to_z() function, which takes as inputs a vector of anthropometric measurements, a vector of ages, a vector of genders, and a uniquely specified chart and measure, and which outputs a vector of Z-scores. In addition, helper functions peditools::recode_von() and peditools::recode_von_xml() are included to easily import VON datasets (exported as a CSV or XML file) into the R environment for analysis.

## Discussion

In this work, we discuss the benefits of developing software tools to perform calculations on LMS-based growth charts and present examples of their utilization in patient care delivery, clinical research, and quality improvement projects.

### Principal Findings and Limitations

#### PediTools Website

The publicly accessible PediTools website [34] makes possible the calculation of exact Z-scores and percentiles for 13 distinct growth charts. Despite the availability of published paper forms of these charts and many of the LMS parameters, there appears to have been an unmet need for publicly available calculators, as demonstrated by PediTools page views increasing to over 500,000 per month. Most visitors (433,438/520,450; 83.28%) are from the United States, but 21 countries had at least 3000 distinct users in the previous year. It is likely that most visitors are health care providers, as inspection of the 100 service provider networks with the largest number of PediTools access sessions in the past year revealed that 52 of the network names contained one of the words health, health care, hospital, or medical. In addition, most email communications to PediTools support have been from dieticians, with some from physicians.

Other than documenting website access statistics, it is difficult to gauge the degree of clinical and research impact of the PediTools website, as before this publication, no citable reference or digital object identifier has been available to allow citation tracking. However, in a nonexhaustive internet search, the PediTools website itself is cited in a number of publications, reviews, and clinical guidelines related to topics such as identifying neonatal and pediatric malnutrition, neurodevelopmental outcomes of preterm newborns, bariatric surgery guidelines, nutrition delivery in chronic disease, and monitoring of postnatal growth in late-preterm newborns [2,49-56]. The combination of website access statistics and citations suggests that the suite of PediTools calculators provides a useful service to practicing clinicians.

PediTools is primarily accessed by users in the United States. It is unclear whether clinicians in other countries use other tools, perhaps localized to their specific populations [57,58]. Alternatively, there may be lack of awareness of the tools’ availability. Dissemination of PediTools has thus far been entirely by word of mouth, and its development has thus far neither been formally presented at conferences nor previously published.

A limitation is that the calculations performed by PediTools are all done server side; therefore, in areas with limited internet availability, the tools are inaccessible. Work is in progress to develop a number of the tools as mobile device apps that do not require internet connectivity, with some preliminary work on iOS now released [59,60].

Another limitation of the PediTools website is that, currently, only charts with LMS-based parameterizations are offered. In some instances, LMS parameterization has been done, but the parameters are not published [61]. Alternative methods of parameterization have also been utilized [62], for example, quantile regression for nomogram generation [63] or fitting a skew t-distribution [64]. The PediTools calculators were implemented in PHP, which works well as a general-purpose scripting language, but it does not generally support more complex statistical calculations. For example, the skew t-distribution does not have a closed form solution, but specialized software in other languages (eg, the GAMLSS package in R) [65] would allow calculation of exact Z-scores, given the model’s 4 parameters (mu, sigma, nu, and tau). A future extension of the PediTools R package could incorporate calculations for charts utilizing different parameterizations.

#### Multisite Comparison of Growth Outcomes in Preterm Infants

The PediTools website analyzes a single patient at a time, as might be appropriate for management of individual patients. Upon applying LMS-based calculations to a large cohort of infants from 7 hospitals in Massachusetts, we were able to characterize the ∆Z weight. Across the overall population, findings included a significant decrease in weight Z-score and an association with larger decreases in Z-score at lower gestational ages. When each of the 7 hospitals was analyzed separately and compared, we found significant interhospital variation in decreases in Z-score and in the degree of association with gestational age. The findings remained similar across different birth year epochs (Figure 5 and data not shown). This observation of growth outcomes is potentially concerning, as growth failure in this vulnerable population is associated with poor neurodevelopmental outcomes, and we show here that the infants at highest risk of poor neurodevelopmental outcome—those born the most preterm—are also at greatest risk of poor growth.

For a number of reasons, the interhospital variations should be taken as a proof of concept and feasibility demonstration of the approach rather than a rigorous comparative analysis of the 7 hospitals. The patient populations of the hospitals differed significantly (Table 3). Although all 7 hospitals participated in the VON registry, which served as the source of the data, participation varied among hospitals, with the Very Low Birth Weight database (401-1500 grams birth weight or 22-29 weeks completed gestation), the Expanded database (all infants admitted to a NICU within 28 days of birth), or even changing participation during the time period of this study. In addition, the discharge disposition varied from 6.4% to 70% transfer to another hospital, versus discharge home. No effort was made to document differences in nutritional practices at each institution. That being said, reanalysis of the dataset restricted to either requiring birth weight<1500 grams or requiring a home discharge disposition did not substantially change any of the findings reported here (data not shown), suggesting that the differences observed were robust to these varied patient populations.

#### Single-Site Growth Outcomes Quality Improvement Project

Assessing outcomes at the hospital level may help identify specific practice differences effective in improving growth, as well as providing a metric to assess and follow performance. In this report, a hospital implemented a bundle of interventions and utilized LMS-based assessment of ∆Z weight from birth to discharge by gestational age at birth to monitor the impact pre- and postintervention. Not only was this method helpful in showing statistically significant changes in improvement in overall growth and reducing the impact of lower gestational age on inferior growth but it was also useful in helping to recognize possible excessive growth (eg, in the most preterm infants in the 2012-2014 epoch).

As a quality improvement project, there was less emphasis on attempting to delineate which specific changes in practice had the greatest impact on outcome, and there was more emphasis on rigorous monitoring of the effect of implementing multiple potentially better practices. We believe that the greatest impact likely came from the consistent, weekly, multidisciplinary review of the ongoing growth of each and every patient in the NICU, as well as ongoing monitoring of neonatal growth as a unit-wide metric. The use of LMS-based calculation of exact Z-scores was critical for this intervention.

A challenge in targeting growth in preterm infants is the lack of evidence conclusively demonstrating exactly what ideal growth should be, but consensus guidelines are emerging [6,49]. Identified indicators of malnutrition include the following: ∆Z over time (with goal ∆Z weight not more negative than −0.8, roughly matching hospital C’s goal after 2014 of ∆Z weight=−0.6), weight gain velocity, actual nutrient intake, days to regain birth weight, length growth velocity, and ∆Z of length for age. A major purpose of the PediTools LMS-based calculators was to make these data easy for clinicians to analyze, track, and understand.

### Comparison With Previous Work

Previous work has analyzed growth of large populations of preterm newborns. Horbar et al [66] drew on data obtained from the full Vermont Oxford Registry on 362,833 newborns born between 2000 and 2013, with birth weight from 501 to 1500 grams. In this large, aggregate population, they reported improvements of growth velocity and a decrease in discharge with growth failure and severe growth failure (defined as discharge at less than the 10th and 3rd percentiles), across the time period from 2000 to 2013. Similarly, Griffin et al [67] reported on 25,899 infants born in California, with birth weight from 500 to 1500 grams or gestational age from 22 to 32 weeks, born between 2005 and 2012. They demonstrated a reduction in fall in weight Z-score between birth and discharge over the time period, as well as a reduction in the proportion of infants discharged home below the 10th percentile for weight or ∆Z weight less than −1. We see similar improvements in less negative ∆Z weight over time, comparing birth years 2006 to 2008 versus 2009 to 2011 (Figure 5).

Although both Horbar et al [66] and Griffin et al [67] report outcomes by birth weight (binned into categorical groups of 250 gram increments and which would therefore include both large for gestation more premature and small for gestation less premature newborns), previous studies have not reported the association described here between growth outcomes as ∆Z weight and gestational age at birth (both continuous variables).

Both of these studies report on the important findings of overall population-level improvement in growth outcomes in preterm newborns across a time period ranging from 2000 to 2013, which likely reflects clinical practice changes across the field of neonatology as a whole, which may result in improved long-term outcomes. However, this information is less helpful for clinicians attempting to assess outcomes at the local hospital level. In addition, the previous studies have not shared the tools needed to make it convenient to perform this analysis on new populations. In fact, without tools to easily assess growth, it is not easy for clinicians to even recognize that there might be an issue with growth outcomes in their patient populations.

A major goal of this study is to make tools available, allowing others to perform their own large-scale growth outcomes analysis, facilitating future research to better describe ideal growth that will lead to optimal long-term outcomes. More than 1200 hospitals around the world participate in the VON registry, and an increasing number of clinical sites use electronic health records from which anthropometric data can be extracted. With this increased availability of growth data, clinicians can easily replicate this analysis in a very small amount of code, using free and open-source tools, including the R statistical programming language, the RStudio integrated development environment, the ggplot2 R visualization package [39-41], and the peditools package described here [44].

### Conclusions

Tools to perform LMS-based growth chart calculations have been made available on a public website and are highly utilized by clinical caregivers worldwide. Applying these methods to a large population of preterm newborns demonstrated widespread overall loss in weight Z-score from birth to discharge; that the magnitude of loss was associated with increasing prematurity, the population at the highest risk of poor neurodevelopment outcomes; and that there was significant interhospital variation in growth outcomes. At 1 site, these tools provided a convenient and reliable outcome measure for a clinical quality improvement project targeting growth. With this report, release of open-source code that implements LMS-based calculations will allow other clinicians and investigators to conveniently perform similar analyses with the promise to improve long-term outcomes in these high-risk pediatric patients.

## Abbreviations

CDC: Centers for Disease Control and Prevention
LMS: lambda-mu-sigma
MIT: Massachusetts Institute of Technology
NICU: neonatal intensive care unit
VON: Vermont Oxford Network
WHO: World Health Organization
∆Z: change in weight Z-score
∆Z weight: change in weight Z-score from birth to discharge
